# Occupational Exposure Profiles and Respiratory Health Outcomes Among Surface and Underground Miners: A Comparative Epidemiological Analysis

**DOI:** 10.3390/ijerph23060805

**Published:** 2026-06-17

**Authors:** Masilu Daniel Masekameni, Thokozane Patrick Mbonane, Khathutshelo Vincent Mphaga, Themba Titus Sigudu, Phoka Caiphus Rathebe

**Affiliations:** 1 Development Studies, School of Social Sciences, University of South Africa, Pretoria 0003, South Africa; 2 Department of Environmental Health, Faculty of Health Sciences, Doornfontein Campus, University of Johannesburg, Johannesburg 2094, South Africamphagakv@gmail.com (K.V.M.); prathebe@uj.ac.za (P.C.R.); 3 Division of Health and Society, School of Public Health, Faculty of Health Sciences, University of the Witwatersrand, Johannesburg 2094, South Africa

**Keywords:** occupational lung disease, mining exposure, chronic cough, dust exposure, Mpumalanga Province

## Abstract

**Highlights:**

**Public health relevance—How does this work relate to a public health issue?**

- Occupational hazardous exposure remains a significant threat to public health.
- Poor occupational health safety culture leads to the prevalence of respiratory illnesses.

**Public health significance—Why is this work of significance to public health?**

- Identification of key exposure profiles is essential in developing occupational health programs.
- Exposure profiles and exposure quantification assist in the implementation of targeted controls to reduce exposure.

**Public health implications—What are the key implications or messages for practitioners, policymakers, and/or researchers?**

- Development of chronic lung diseases leads to households income loss and reduced life expectancy.
- Workers in underground mines are significantly exposed to elevated dust levels compared to surface mine workers.

**Abstract:**

Occupational lung diseases remain a significant public health concern in mining populations, particularly in high-exposure environments. This study examined occupational exposure profiles and respiratory health outcomes among surface and underground miners in Mpumalanga Province. A cross-sectional analytical design was employed among 239 mine workers. Data on socio-demographic characteristics, occupational exposures, behavioural factors, and respiratory outcomes were analysed using descriptive statistics, chi-square tests, and logistic regression models. Underground miners were significantly more likely to report high dust exposure (44.9% vs. 24.1%), poor ventilation (60.6% vs. 39.3%), and longer working hours (>8 h: 68.5% vs. 50.0%) compared to surface miners. They also reported a higher prevalence of respiratory symptoms, including chronic cough (45.7% vs. 25.9%), shortness of breath (41.7% vs. 23.2%), wheezing (34.6% vs. 18.8%), and diagnosed lung disease (23.6% vs. 9.8%). Multivariable analysis showed that underground mining (AOR = 1.92; 95% CI: 1.08–3.41), smoking (AOR = 1.78; 95% CI: 1.02–3.11), and high dust exposure (AOR = 2.89; 95% CI: 1.45–5.76) were independent predictors of chronic cough. A significant interaction between smoking and underground mining (AOR = 2.74; 95% CI: 1.32–5.68) further amplified respiratory risk. Additionally, underground miners demonstrated lower levels of knowledge (48.8% vs. 66.1%) and poorer preventive practices (44.1% vs. 64.3%). These findings highlight the combined influence of occupational and behavioural factors on respiratory health and highlight the need for integrated interventions to reduce the burden of occupational lung diseases.

## 1. Introduction

Occupational lung diseases remain a major global public health concern, particularly in low-and middle-income countries where mining continues to be a key economic activity [1,2]. Despite advances in industrial hygiene and regulatory frameworks, workers in the mining sector are still exposed to hazardous airborne particulates, including silica dust, coal dust, and other respirable materials that contribute to the development of chronic respiratory conditions such as pneumoconiosis, chronic obstructive pulmonary disease (COPD), and silicosis [3,4,5]. These conditions are largely preventable, yet they continue to impose a substantial burden on workers, healthcare systems, and national economies [6,7].

The epidemiology of occupational lung diseases has evolved, with increasing recognition of the complex interplay between environmental exposures, occupational conditions, and individual behavioural risk factors [8,9]. In mining environments, particularly underground settings, workers are often exposed to higher concentrations of respirable dust due to confined working spaces, inadequate ventilation, and prolonged exposure durations [10,11]. These structural and environmental factors significantly elevate the risk of respiratory morbidity compared to surface mining operations [12]. Evidence consistently shows that underground miners experience a higher prevalence of chronic cough and lung function impairment than surface workers [13,14].

In addition to occupational exposures, behavioural factors such as cigarette smoking play a critical role in exacerbating respiratory health outcomes. Smoking is a well-established independent risk factor for chronic respiratory disease and may act synergistically with occupational dust exposure to accelerate lung damage [15,16]. This interaction is particularly relevant in high-risk environments such as underground mining, where cumulative exposures may significantly amplify adverse health effects [17].

Furthermore, knowledge and adherence to preventive practices, such as the consistent use of personal protective equipment (PPE), are essential components of occupational health and safety. However, evidence suggests persistent gaps in awareness and compliance among mine workers, particularly in resource-constrained settings [18]. These deficiencies may contribute to the continued occurrence of preventable occupational lung diseases and highlight the need for integrated interventions addressing both environmental and behavioural determinants [19].

In South Africa, and particularly in Mpumalanga Province, mining remains a dominant industry with a long-standing burden of occupational respiratory diseases, including pneumoconiosis and silica-related lung conditions [20]. The persistence of these diseases highlights the need for context-specific epidemiological studies to better understand exposure patterns and inform targeted prevention strategies.

Analysis of the current dataset demonstrates significant differences in occupational exposure profiles and respiratory health outcomes between surface and underground miners. Underground workers experienced higher levels of dust exposure, poorer ventilation, longer working hours, and lower adherence to personal protective equipment, alongside a significantly greater burden of chronic cough and diagnosed lung disease. Additionally, smoking and inconsistent PPE use were identified as key determinants of chronic cough, with evidence of a significant interaction between smoking and underground mining environments, suggesting a synergistic amplification of respiratory risk.

This study compared occupational exposure profiles and respiratory health outcomes among surface and underground miners in Mpumalanga Province, South Africa. Specifically, the study was designed to test the hypothesis that occupational exposure profiles and respiratory health outcomes differ between surface and underground miners’ chronic cough.

By integrating environmental, behavioural, and occupational determinants, this study contributes to the modern epidemiology of occupational lung diseases and provides evidence to inform targeted workplace interventions and policy development in high-risk mining populations.

### 1.1. Study Design and Setting

This study employed a quantitative cross-sectional analytical design to investigate occupational exposure profiles, respiratory health outcomes, and their association with behavioural and awareness-related factors among mine workers in Mpumalanga Province. The study specifically compared surface and underground mining environments to better understand differences in exposure intensity and health outcomes.

The study was conducted at Syferfontein and Shondoni mining operations, two major mining sites in the province. Mpumalanga Province is one of South Africa’s key mining regions, characterised by extensive coal mining activities and a well-documented burden of occupational respiratory diseases. Inclusion of participants from both surface and underground operations allowed for comparative analysis across different occupational environments, thereby providing a comprehensive understanding of workplace exposures, health awareness, and respiratory health risks among miners. Workers included in this study were not assigned to surface or underground operations by the investigators. Rather, mining type reflected participants’ existing occupational assignments within the participating mines. Allocation to surface or underground operations is determined by mine management according to operational requirements, job specialization, technical competencies, training, experience, and workforce needs. Surface workers are generally involved in coal handling, transportation, equipment maintenance, processing plant operations, stockpile management, and administrative support activities. In contrast, underground workers are primarily involved in coal extraction, drilling, roof support installation, haulage, and equipment operation within confined underground environments. Underground operations often require specialised skills, familiarity with underground working conditions, and experience with mining equipment and safety procedures. Consequently, workers employed underground may accumulate longer periods of service within these roles, which may partly explain the longer tenure observed among underground workers in this study. Workers were classified according to their current work assignment as either surface or underground miners. Assignment to these work areas is determined by operational requirements, job responsibilities, training, and work experience. Although workers may occasionally be transferred between sections during their employment, information regarding previous work locations and workers’ knowledge of conditions in alternative mining environments was not collected. Therefore, occupational exposure assessments were based on workers’ current work environments and self-reported experiences.

Surface and underground mining operations differ substantially in their work activities and environmental conditions. Surface workers are generally involved in coal handling, transportation, equipment maintenance, processing plant operations, stockpile management, and other support activities conducted in open-air environments. Underground workers primarily perform coal extraction, drilling, roof support installation, haulage, and equipment operation within confined subterranean spaces. Underground mining environments typically rely on mechanical ventilation systems to control airborne contaminants and are characterised by reduced natural airflow, whereas surface operations benefit from greater natural ventilation and dispersion of airborne dust. Worker allocation to surface or underground operations is determined by operational requirements, job specialization, technical competencies, and work experience. These inherent differences in occupational environments provided the rationale for comparing exposure profiles and respiratory health outcomes between the two mining groups.

Surface and underground mining operations differ substantially in their work activities and environmental conditions. Surface workers are generally involved in coal handling, transportation, equipment maintenance, processing operations, stockpile management, and support activities conducted in open-air environments. Underground workers primarily perform coal extraction, drilling, haulage, roof support installation, and equipment operation within confined subterranean spaces. Underground mining environments typically rely on mechanical ventilation systems and are characterised by reduced natural airflow, whereas surface operations benefit from greater natural ventilation and dispersion of airborne contaminants.

### 1.2. Sampling Technique and Study Population

A stratified random sampling technique was employed to select participants from a combined workforce of approximately 500 employees at the participating mining operations. Stratification was based on mining type (surface and underground) to ensure adequate representation of both occupational environments. Employee lists provided by mine management served as the sampling frame, and eligible workers within each stratum were selected using a simple random sampling (lottery) method. Following random selection, participation was voluntary, and written informed consent was obtained from all participants prior to data collection. Mining type was determined by workers’ existing occupational assignments at the participating mines and was not allocated by the investigators. Within each stratum, participants were selected using a simple random sampling (lottery) method, thereby ensuring that each eligible worker had an equal probability of inclusion.

The sample size was calculated using Epi Info version 3 because of its dedicated epidemiological sample size calculation module. Statistical analyses were subsequently performed using STATA version 20 (StataCorp, College Station, TX, USA), which was used for data management, descriptive analyses, bivariate analyses, and multivariable logistic regression modelling. The use of separate software packages reflected software functionality and researcher preference and did not affect the validity of the analyses or study findings. In the absence of reliable local prevalence estimates for occupational chronic cough and exposure characteristics among miners in the study setting, a prevalence of 50% was assumed. This conservative approach maximises sample size requirements and is recommended when prior prevalence estimates are unavailable. A 95% confidence level, 5% margin of error, and design effect of 1 were applied, yielding a minimum sample size of 218 participants. The assumption of a 50% prevalence corresponds to the maximum variance scenario (*p* = 0.5), which provides the most conservative sample size estimate when prior prevalence data are unavailable. Eligible participants included mine workers aged 18 years and above who were currently employed in either surface or underground mining operations at the selected sites. This study included participants across varying levels of work experience to capture a comprehensive range of occupational exposure profiles. Workers with missing information on the primary exposure variable (mining type) or on key occupational exposure variables (dust exposure level, ventilation quality, exposure duration, and PPE use) or respiratory outcome variables (chronic cough, shortness of breath, wheezing, and diagnosed lung disease) were excluded from the final analysis. These variables were essential for the comparative and multivariable analyses undertaken in the study.

### 1.3. Questionnaire Development and Data Collection

Data were collected using a structured questionnaire designed to obtain information on socio-demographic characteristics, occupational exposures, behavioural factors, respiratory health outcomes, knowledge, and preventive practices. The questionnaire was developed in English and informed by peer-reviewed literature on occupational lung diseases, pneumoconiosis risk factors, and relevant occupational health and safety frameworks, including the Occupational Health and Safety Act, 1993 (Act 85 of 1993). Additional items were adapted from established research tools and reports focusing on mining health and safety in South Africa.

The instrument comprised five sections: (A) socio-demographic characteristics, including age, sex, and education level; (B) clinical and chronic cough; (C) occupational exposure hazards; (D) knowledge of occupational health and safety; and (E) preventive practices and awareness. The questionnaire was designed to be clear, concise, and appropriate for the study population.

Information collected included socio-demographic characteristics, behavioural factors such as smoking status, and occupational history including job type and work experience. Occupational exposure variables assessed included self-reported dust exposure level (low, moderate, high), duration of daily exposure (≤8 h, >8 h), ventilation quality (adequate, poor), and use of personal protective equipment (PPE) (consistent, inconsistent). PPE use was assessed based on participants’ self-reported consistency of PPE utilisation during routine work activities. Although PPE use is mandated by workplace occupational health and safety policies and compliance is generally monitored by supervisors, the variable measured in this study reflected workers’ reported PPE utilisation behaviour rather than organisational PPE requirements or supervisory enforcement practices. Occupational exposure variables were assessed using self-reported measures. Participants were asked to classify their typical dust exposure level as low, moderate, or high based on the perceived frequency and intensity of dust encountered during routine work activities. Ventilation quality was assessed based on participants’ perceptions of airflow and air quality in their work environment and categorised as adequate or poor. Exposure duration was classified as ≤8 h or >8 h per working day, while PPE use was categorised as consistent or inconsistent according to self-reported adherence during routine work activities. As objective environmental measurements were not available, these variables represent subjective assessments of occupational exposure conditions. In underground mining operations, ventilation is typically maintained through mechanical systems comprising ventilation shafts, intake and return airways, main and auxiliary fans, and associated ventilation controls designed to provide fresh air and remove airborne contaminants. Surface mining environments generally benefit from natural air circulation. In this study, ventilation quality was assessed through workers’ self-reported perceptions and was categorised as adequate or poor rather than being based on direct engineering measurements of airflow or air quality.

Respiratory health outcomes were assessed using self-reported symptoms and clinical history, including chronic cough, shortness of breath, wheezing, and prior diagnosis of lung disease. In addition, knowledge and preventive practices related to occupational lung diseases were evaluated using a composite scoring system, with knowledge categorised as good (≥80%) or poor (<80%), and preventive practices classified as adequate or inadequate.

A pilot study was conducted prior to the main study using approximately 10% of the target population to assess the clarity, validity, and reliability of the instrument. Internal consistency was evaluated using Cronbach’s alpha coefficient, which yielded a value of 0.81, indicating good reliability of the questionnaire. Several measures were implemented to minimise reporting bias. The questionnaire used standardised questions and separated occupational exposure items from respiratory health outcome items. Participants were assured of confidentiality and anonymity to encourage accurate reporting and reduce socially desirable responses. A pilot study was conducted to improve clarity and consistency of questionnaire items prior to data collection.

### 1.4. Study Variables

The primary outcome variable in this study was chronic cough, operationalised as the presence of chronic cough. Chronic cough was treated as a binary variable (yes/no) and served as the main dependent variable in both bivariate and multivariable analyses, given its relevance as an early and sensitive indicator of occupational lung disease.

Secondary outcome variables included other clinically relevant respiratory conditions and symptoms, namely shortness of breath, wheezing, and self-reported diagnosed lung disease. These variables were also analysed as binary outcomes (presence/absence) and were used to provide a broader assessment of respiratory health status among miners.

The main independent variable was mining type, categorised as surface or underground, reflecting differences in occupational environments and exposure intensity. This variable was central to the comparative objectives of the study.

A range of socio-demographic variables were included as potential confounders and effect modifiers. These comprised age group (18–35, 36–45, ≥46 years), sex (male/female), and education level (≤high school, tertiary). These variables were selected based on their known association with occupational risk exposure and health outcomes.

Behavioural factors included smoking status (smoker/non-smoker), which was examined both as an independent predictor and as part of an interaction term with mining type to assess potential effect modification on respiratory outcomes.

Occupational variables constituted key explanatory factors in this study and captured multiple dimensions of workplace exposure. Work experience was categorised as <5 years, 5–10 years, and >10 years to reflect cumulative exposure over time. Dust exposure level was classified as low, moderate, or high based on self-reported exposure intensity, while exposure duration was measured in terms of daily working hours (≤8 h and >8 h). Workplace environmental conditions were assessed through ventilation quality, categorised as adequate or poor. In addition, adherence to preventive measures was evaluated through the use of personal protective equipment (PPE), classified as consistent or inconsistent. Together, these variables provided a comprehensive assessment of occupational risk factors associated with respiratory health outcomes.

In addition, knowledge and preventive practice variables were assessed using composite scores derived from questionnaire responses. Knowledge of occupational lung diseases was categorised as good (≥80%) or poor (<80%), while preventive practices were classified as adequate or inadequate based on predefined scoring thresholds. These variables were included to evaluate the role of awareness and behaviour in mitigating occupational risk.

All variables were selected based on epidemiological relevance and existing literature on occupational lung diseases, and were included in the analysis to assess their independent and combined effects on respiratory health outcomes.

### 1.5. Data Management and Statistical Analysis

The sample size was calculated using Epi Info version 3 because of its dedicated epidemiological sample size calculation module. Collected data were first entered into Microsoft Excel and subsequently transferred to STATA (StataCorp, College Station, TX, USA) for data management and statistical analysis. The use of separate software packages reflected software functionality and researcher preference and did not affect the validity of the analyses or study findings.

Data were analysed using standard statistical procedures. Descriptive statistics were used to summarise participant characteristics, occupational exposures, and health outcomes, with categorical variables presented as frequencies and percentages. Differences between surface and underground miners were assessed using the chi-square test.

Bivariate logistic regression analysis was conducted to examine associations between independent variables and chronic cough, with results reported as crude odds ratios (ORs) and 95% confidence intervals (CIs). Variables identified a priori as potential confounders based on epidemiological evidence and previous literature, including age group, sex, smoking status, and work ex-perience, were retained in the multivariable model regardless of their statistical significance in the bivariate analyses. Additional variables associated with chronic cough at *p* < 0.20 in the bivariate analyses were considered for inclusion in the multivariable model. This approach ensured adequate adjustment for known confounding factors while allowing potentially important explanatory variables to be evaluated.

Multivariable logistic regression analysis was performed to identify independent predictors of chronic cough, with adjusted odds ratios (AORs) and 95% confidence intervals reported. The model adjusted for age group, sex, education level, smoking status, work experience, dust exposure level, use of personal protective equipment (PPE), ventilation quality, and exposure duration. Statistical significance was assessed using a two-sided alpha level of 0.05. Actual *p*-values are reported throughout the manuscript, with smaller *p*-values indicating stronger statistical evidence against the null hypothesisTo assess effect modification, an interaction term between smoking status and mining type was included in the regression model. The significance of this interaction term was evaluated to determine whether the effect of smoking on respiratory outcomes differed between surface and underground miners.

### 1.6. Ethical Considerations

This study was conducted in line with the ethical principles of the Declaration of Helsinki, ensuring respect for participants’ dignity, autonomy, and overall well-being throughout the research process. Ethical approval was obtained from the Research Ethics Committee of the Faculty of Health Sciences at the University of Johannesburg (REC-2816-2024) on the 12 June 2024. Prior to the initiation of the study, and all study procedures involving human participants were reviewed and approved to ensure adherence to established ethical standards.

In addition to institutional approval, formal permission to conduct the study was secured from the management of the participating mining sites, Syferfontein and Shondoni. Engagement with site management ensured that the study complied with organisational regulations and was conducted without disrupting normal operations or compromising worker safety.

Participation was entirely voluntary, and all participants provided written informed consent after being fully informed about the study objectives, procedures, potential risks, and benefits. Participants were also informed of their right to withdraw from the study at any time without any adverse consequences. To maintain confidentiality and anonymity, personal identifiers were replaced with unique alphanumeric codes. All data were stored securely on password-protected systems, accessible only to the research team, ensuring the protection of participant information throughout the study.

## 2. Results

### 2.1. Socio-Demographic Characteristics of Study Participants by Mining Type

Table 1 presents the socio-demographic profile of 239 mine workers stratified by mining type (surface vs. underground) in Mpumalanga Province.

Overall, the study population was predominantly within the economically active age range, with the largest proportion aged 36–45 years (38.5%), followed by 18–35 years (32.6%) and those aged ≥46 years (28.9%). There was a statistically significant difference in age distribution between surface and underground miners (*p* = 0.041). Surface miners were relatively younger, with a higher proportion in the 18–35 age group (37.5%), whereas underground miners were more represented in the 36–45 age group (41.7%). This suggests a potential age-related occupational sorting, where more experienced or older workers may be concentrated in underground operations.

The workforce was overwhelmingly male (82.8%), reflecting the gendered nature of the mining sector. Although a higher proportion of males was observed among underground miners (86.6%) compared to surface miners (78.6%), this difference was not statistically significant (*p* = 0.098). Female participation remained low across both groups.

In terms of educational attainment, the majority of participants had a high school level education or less (59.8%), with a smaller proportion having tertiary education (40.2%). While underground miners had a slightly higher proportion of tertiary education (44.1%) compared to surface miners (35.7%), this difference was not statistically significant (*p* = 0.182), indicating comparable educational profiles across mining types.

Smoking status differed significantly by mining type (*p* = 0.027). A higher proportion of underground miners were smokers (48.8%) compared to surface miners (34.8%), whereas non-smoking was more prevalent among surface workers (65.2%). This finding is particularly important given the synergistic effect of smoking and occupational dust exposure on respiratory health outcomes.

Work experience also showed a statistically significant association with mining type (*p* = 0.015). Surface miners were more likely to have less than 5 years of experience (42.9%), whereas underground miners had a higher proportion of workers with longer experience, particularly those with more than 10 years (36.2%). This pattern reinforces the observation that underground mining may be associated with a more experienced workforce. Worker assignment to underground operations is generally influenced by operational requirements, job specialization, and accumulated work experience. This may partly explain the higher proportion of experienced workers observed in underground mining environments.

### 2.2. Occupational Exposure Profiles in Surface and Underground Mining Activity

**Figure 1:**
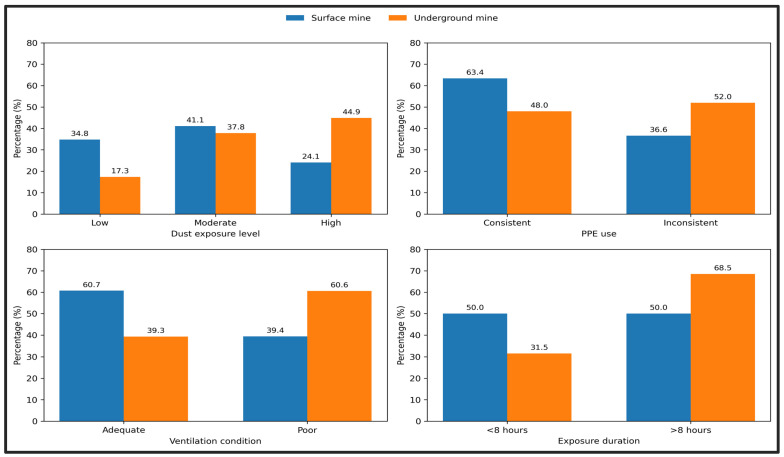
Occupational exposure profiles by mining type.

Figure 1 summarises the occupational exposure characteristics of mine workers stratified by mining type. Overall, the findings reveal marked and statistically significant differences in exposure intensity and workplace conditions between surface and underground miners.

Dust exposure levels varied across mining types. While the largest proportion of participants reported moderate exposure (39.3%), underground miners were disproportionately exposed to high dust levels (44.9%) compared to surface miners (24.1%). Conversely, low dust exposure was more common among surface workers (34.8%) than underground workers (17.3%). This pattern highlights the elevated environmental hazard associated with underground mining operations, where confined spaces and limited dispersion contribute to higher particulate concentrations.

Personal protective equipment (PPE) use also differed significantly by mining type. Overall, just over half of the participants reported consistent PPE use (55.2%). However, adherence was notably higher among surface miners (63.4%) compared to underground miners (48.0%). In contrast, inconsistent PPE use was more prevalent among underground workers (52.0%), raising concerns about inadequate protection in settings where exposure risk is greatest.

Ventilation quality showed a similarly visible disparity. Adequate ventilation was reported by 60.7% of surface miners but only 39.4% of underground miners. Poor ventilation conditions were markedly more common in underground environments (60.6%) compared to surface settings (39.3%). Given the role of ventilation in mitigating airborne dust exposure, this finding highlights a critical structural risk factor contributing to occupational lung disease among underground workers.

Exposure duration further differentiated the two groups. A greater proportion of underground miners reported working more than 8 h per day (68.5%) compared to surface miners (50.0%). In contrast, shorter working hours (≤8 h) were more common among surface workers (50.0%) than underground workers (31.5%). Prolonged exposure duration among underground miners likely compounds the cumulative risk of respiratory morbidity.

### 2.3. Self-Reported Respiratory Health Outcomes

**Figure 2:**
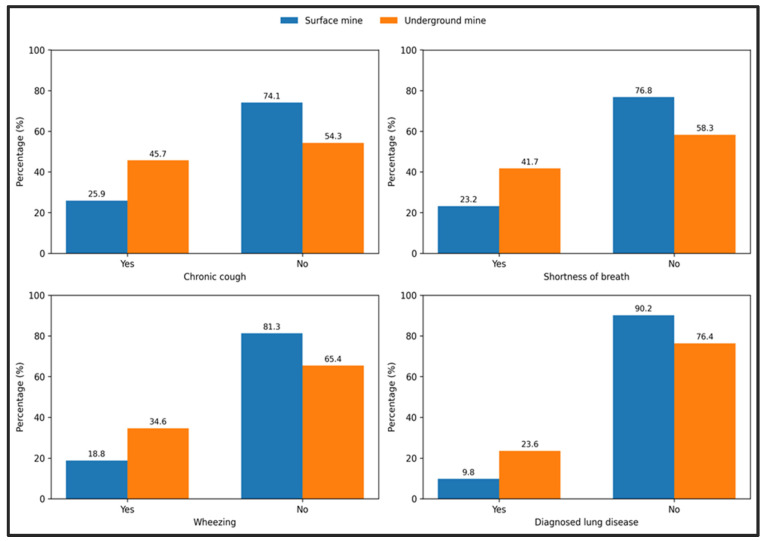
Respiratory health outcomes among miners.

Figure 2 presents the distribution of self-reported respiratory health outcomes among miners, stratified by mining type. Overall, the findings indicate a significantly higher burden of chronic cough and diagnosed lung disease among underground miners compared to their surface counterparts.

Chronic cough was reported by 36.4% of all participants, with a statistically significant difference between mining types (*p* = 0.002). The prevalence was substantially higher among underground miners (45.7%) compared to surface miners (25.9%), suggesting increased exposure to respiratory irritants in underground environments.

Similarly, shortness of breath was reported by 33.1% of participants and differed significantly by mining type (*p* = 0.003). Underground miners again exhibited a higher prevalence (41.7%) compared to surface workers (23.2%). This pattern is consistent with impaired respiratory function potentially associated with prolonged exposure to dust and inadequate ventilation.

Wheezing was reported by 27.2% of the study population and also showed a varied difference between groups. A higher proportion of underground miners reported wheezing (34.6%) compared to surface miners (18.8%), further reinforcing the elevated respiratory risk associated with underground mining conditions.

In terms of clinically diagnosed lung disease, 17.2% of participants reported a diagnosis, with a significantly higher prevalence among underground miners (23.6%) compared to surface miners (9.8%). This finding suggests not only a higher burden of symptoms but also a greater likelihood of confirmed respiratory pathology among underground workers.

### 2.4. Bivariate Analysis of Factors Associated with Chronic Cough

Table 2 presents the results of the bivariate analysis examining factors associated with chronic cough among mine workers. The findings indicate several significant occupational and behavioural determinants of chronic cough.

Mining type was significantly associated with chronic cough (*p* = 0.002). Underground miners had more than twice the odds of reporting chronic cough compared to surface miners (OR = 2.38; 95% CI: 1.39–4.07). This highlights the increased respiratory risk associated with underground mining environments, likely due to higher dust exposure and poorer ventilation conditions.

Smoking status was also a significant predictor (*p* = 0.005). Smokers had approximately two times higher odds of experiencing chronic cough compared to non-smokers (OR = 2.11; 95% CI: 1.25–3.56). This finding highlights the well-established role of tobacco use as an important independent and potentially synergistic risk factor in occupational lung disease.

Dust exposure demonstrated a strong dose–response relationship with chronic cough. While moderate exposure was associated with increased odds (OR = 1.67; 95% CI: 0.89–3.12), this association was not statistically significant (*p* = 0.108). In contrast, high dust exposure was significantly associated with chronic cough (OR = 3.45; 95% CI: 1.83–6.49; *p* < 0.001), indicating that workers exposed to high dust levels were more than three times as likely to report symptoms compared to those with low exposure. This gradient effect provides strong epidemiological evidence of exposure–outcome linkage.

Personal protective equipment (PPE) use was another significant factor (*p* = 0.013). Workers who reported inconsistent PPE use had nearly twice the odds of chronic cough compared to those who used PPE consistently (OR = 1.94; 95% CI: 1.15–3.27). This suggests that adherence to protective measures plays a critical role in mitigating respiratory risk.

### 2.5. Multivariable Logistic Regression Analysis of Predictors of Chronic Cough

Table 3 presents the results of the multivariable logistic regression model assessing independent predictors of chronic cough among mine workers. After adjusting for relevant socio-demographic, occupational, and behavioural factors, several variables remained significantly associated with the outcome.

Mining type emerged as an independent predictor of chronic cough (*p* = 0.026). Underground miners had significantly higher odds of reporting chronic cough compared to surface miners (AOR = 1.92; 95% CI: 1.08–3.41). This finding confirms that the elevated risk observed in the bivariate analysis persists even after controlling for other covariates, underscoring the inherent occupational hazards associated with underground mining environments.

Smoking status also remained significantly associated with chronic cough (*p* = 0.041). Smokers had approximately 1.8 times higher odds of experiencing chronic cough compared to non-smokers (AOR = 1.78; 95% CI: 1.02–3.11). This highlights the independent contribution of tobacco use to respiratory morbidity, beyond occupational exposures.

Dust exposure demonstrated a clear exposure–response relationship in the adjusted model. While moderate exposure was not statistically significant (AOR = 1.41; 95% CI: 0.73–2.72; *p* = 0.305), high dust exposure remained a strong and significant predictor of chronic cough (AOR = 2.89; 95% CI: 1.45–5.76; *p* = 0.002). Workers exposed to high dust levels were nearly three times more likely to report symptoms compared to those with low exposure, reinforcing the central role of dust inhalation in the pathogenesis of occupational lung diseases.

Inconsistent use of personal protective equipment (PPE) was associated with increased odds of chronic cough (AOR = 1.63; 95% CI: 0.94–2.83), although this association did not reach statistical significance (*p* = 0.081). Similarly, longer work experience (≥5 years) showed elevated odds (AOR = 1.56; 95% CI: 0.89–2.73), but this was also not statistically significant (*p* = 0.118). Despite the lack of statistical significance, both variables demonstrate clinically relevant trends that may warrant further investigation in larger studies.

### 2.6. Distribution of Knowledge Levels and Preventive Practices Regarding Occupational Lung Diseases

**Figure 3:**
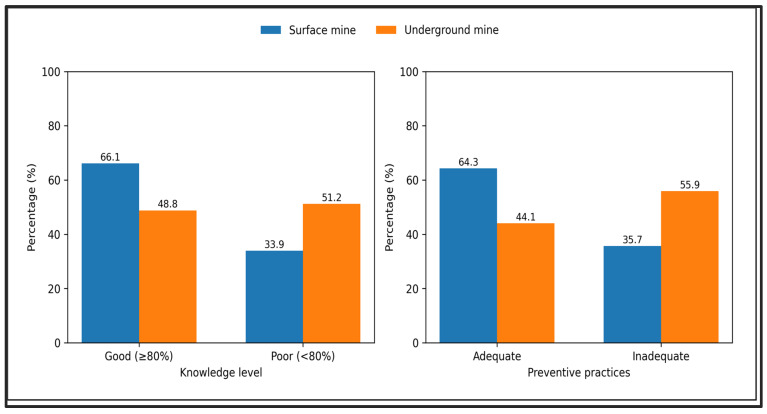
Knowledge and preventive practices related to occupational lung diseases.

Figure 3 presents the distribution of knowledge levels and preventive practices regarding occupational lung diseases among miners, stratified by mining type. The findings reveal significant disparities between surface and underground workers in both domains.

Overall, just over half of the participants demonstrated good knowledge of occupational lung diseases (56.9%), while 43.1% had poor knowledge. There was a difference in knowledge levels by mining type. Surface miners were more likely to have good knowledge (66.1%) compared to underground miners (48.8%). Conversely, poor knowledge was more prevalent among underground workers (51.2%) than surface workers (33.9%). This suggests potential gaps in health education and awareness, particularly among those working in higher-risk underground environments.

A similar pattern was observed for preventive practices. Slightly more than half of the participants reported adequate preventive practices (53.6%), while 46.4% demonstrated inadequate practices. Surface miners reported higher levels of adequate preventive practices (64.3%) compared to underground miners (44.1%). In contrast, inadequate practices were more common among underground workers (55.9%) than surface workers (35.7%).

### 2.7. Effects of Smoking and Mining Type on Respiratory Outcomes

Table 4 presents the interaction effects between smoking status and mining type on chronic respiratory outcomes. The analysis demonstrates a significant effect modification by mining environment.

The interaction between smoking and underground mining was statistically significant (AOR = 2.74; 95% CI: 1.32–5.68; *p* = 0.007), indicating that smokers working underground had nearly three times higher odds of experiencing chronic cough compared to the reference group. This finding suggests a synergistic effect, where the combined exposure to tobacco smoke and high-intensity occupational hazards, such as elevated dust levels and poor ventilation, substantially amplifies respiratory risk.

In contrast, the interaction between smoking and surface mining was not statistically significant (AOR = 1.45; 95% CI: 0.72–2.91; *p* = 0.298). Although the direction of association indicates increased odds, the lack of statistical significance suggests that the effect of smoking alone in lower-exposure surface environments may be less pronounced or insufficient to produce a measurable combined effect.

## 3. Discussion

This study provides important insights into the modern epidemiology of occupational lung diseases by demonstrating significant differences in exposure profiles and respiratory health outcomes between surface and underground miners in Mpumalanga Province. The findings highlight the multifactorial nature of respiratory morbidity in mining populations, driven by a combination of occupational exposures, behavioural risk factors, and workplace conditions. The observed differences between mining types should be interpreted within the context of distinct occupational roles and workplace environments. Underground miners typically perform extraction and haulage activities in enclosed settings with limited natural ventilation, whereas surface workers operate in more open environments where airborne contaminants are more readily dispersed. These structural differences likely contribute to the higher exposure levels observed among underground workers.

The longer tenure observed among underground miners may reflect differences in workforce allocation and job specialization within the participating mines. Underground operations often require workers with specific technical skills, operational experience, and familiarity with underground working conditions. As a result, employees assigned to underground operations may remain in these roles for longer periods, contributing to the observed differences in work experience between mining environments.

A key finding of this study is the substantially higher burden of chronic cough and diagnosed lung disease among underground miners. This aligns with existing evidence indicating that underground mining environments are associated with higher concentrations of respirable dust due to confined spaces and limited ventilation [21,22]. The observed higher prevalence of chronic cough, shortness of breath, and wheezing among underground workers is consistent with studies demonstrating increased respiratory impairment and lung function decline in similar occupational settings [23,24]. These findings reinforce the well-established link between cumulative dust exposure and respiratory morbidity.

The study further identified a strong exposure–response relationship between dust exposure and chronic cough, with high dust exposure significantly increasing the odds of chronic cough. This finding is supported by epidemiological studies showing that prolonged exposure to respirable crystalline silica and coal dust is a major determinant of pneumoconiosis and other chronic respiratory conditions [25,26]. The lack of statistical significance for moderate exposure suggests a possible threshold effect, where adverse health outcomes become more pronounced at higher exposure levels.

Smoking emerged as an independent predictor of chronic cough, consistent with extensive literature documenting its role in respiratory disease pathogenesis [27,28]. Importantly, the interaction analysis revealed a significant synergistic effect between smoking and underground mining, indicating that combined exposures substantially amplify respiratory risk. This interaction has been reported in previous studies, where smoking exacerbates the harmful effects of occupational dust exposure, leading to accelerated lung function decline and increased disease severity [29,30]. These findings highlight the need for integrated workplace health interventions that address both occupational and behavioural risk factors.

Although inconsistent use of personal protective equipment (PPE) did not reach statistical significance in the adjusted model, it was associated with increased odds of chronic cough. This is consistent with evidence suggesting that proper and consistent use of respiratory protective equipment can significantly reduce inhalation of hazardous particles [31]. The lower adherence to PPE observed among underground miners in this study is particularly concerning, given their higher exposure levels, and may reflect barriers such as discomfort, inadequate training, or limited enforcement of safety protocols [32].

Workplace conditions, including ventilation and exposure duration, also play a critical role in shaping respiratory health outcomes. The study found that underground miners were more likely to experience poor ventilation and longer working hours, both of which contribute to increased cumulative exposure. Similar findings have been reported in occupational health research, where inadequate ventilation systems and extended exposure durations are key determinants of dust-related lung disease [33,34]. These structural factors highlight the importance of engineering controls and regulatory oversight in reducing occupational health risks.

Another important finding of this study relates to disparities in knowledge and preventive practices. Underground miners demonstrated lower levels of knowledge and poorer adherence to preventive measures compared to surface workers. This is consistent with studies showing that limited awareness and inadequate health education contribute to suboptimal safety practices in high-risk occupational settings [35]. Improving worker knowledge through targeted education and training programmes has been shown to enhance compliance with safety measures and reduce exposure risks [36].

The findings of this study have important implications for occupational health policy and practice. First, they emphasise the need for strengthened dust control measures, particularly in underground mining environments. Second, they highlight the importance of integrating smoking cessation programmes into workplace health initiatives. Third, they highlight the need for improved enforcement of PPE use and enhanced worker training. Finally, the observed interaction between smoking and occupational exposure suggests that risk assessments should consider combined exposures rather than treating risk factors in isolation.

Despite its strengths, this study has several limitations that should be considered when interpreting the findings. First, the cross-sectional design precludes the establishment of temporal or causal relationships between occupational exposures and respiratory health outcomes. Second, both occupational exposure variables and respiratory health outcomes were obtained through participant self-reports, introducing the possibility of common method bias (common source bias). Participants who perceived themselves to be highly exposed may have been more likely to report chronic cough, potentially inflating the observed associations between exposures and outcomes. The use of self-reported information also introduces the potential for reporting bias and exposure misclassification, particularly for variables such as dust exposure level, ventilation quality, exposure duration, and personal protective equipment use. Furthermore, occupational exposures were not verified using objective environmental monitoring data, such as airborne dust measurements or workplace ventilation assessments. Similarly, respiratory health outcomes were not clinically verified through medical examinations, lung function testing, radiological investigations, or occupational health records, and therefore may be subject to outcome misclassification. Although a structured questionnaire, pilot testing, and multivariable adjustment for important confounders were used to improve data quality and minimise bias, residual measurement error cannot be excluded. Finally, the study was conducted in only two mining operations in Mpumalanga Province, which may limit the generalisability of the findings to other mining settings. Future longitudinal studies incorporating objective environmental exposure measurements, industrial hygiene assessments, spirometry, and clinical evaluation of respiratory outcomes are recommended to validate these findings and provide a more comprehensive understanding of the relationship between occupational exposures and respiratory health among mine worker’s chronic cough. In conclusion, this study contributes to the growing body of evidence on the modern epidemiology of occupational lung diseases by demonstrating that underground miners experience significantly higher exposure levels and a greater burden of respiratory morbidity. The findings highlight the importance of addressing both environmental and behavioural risk factors through comprehensive and integrated occupational health interventions.

## 4. Conclusions

This study provides compelling evidence of significant disparities in occupational exposure profiles and respiratory health outcomes between surface and underground miners in Mpumalanga Province. Underground miners were consistently exposed to higher levels of occupational risk, including elevated dust exposure, poorer ventilation, longer working hours, and lower adherence to personal protective measures. These adverse conditions were reflected in a substantially higher burden of chronic cough and diagnosed lung disease among this group.

The findings further demonstrate that occupational lung disease in this population is driven by a combination of environmental and behavioural factors. High dust exposure and underground mining independently increased the risk of chronic cough, while smoking significantly exacerbated this risk. The observed interaction between smoking and underground mining highlights the importance of considering combined exposures in occupational health risk assessments.

In addition, the study identified critical gaps in knowledge and preventive practices, particularly among underground miners who are most at risk. These gaps likely contribute to ongoing exposure and disease burden, underscoring the need for strengthened occupational health education and behavioural interventions.

Overall, this study contributes to the modern epidemiology of occupational lung diseases by providing context-specific evidence from a high-risk mining population. The findings highlight the urgent need for comprehensive and integrated occupational health strategies that include improved dust control, enhanced ventilation, strict enforcement of PPE use, and targeted smoking cessation programmes. Addressing these factors is essential to reducing the burden of respiratory disease and improving the health and safety of mine workers.

Future research should focus on longitudinal assessments and incorporate objective measures of exposure and lung function to better understand causal relationships and inform more effective prevention strategies.

## Figures and Tables

**Table 1:** Socio-demographic Distribution of Study Participants Across Mining Categories.

Variable	Category	Total (n = 239)		Surface (n = 112)		Underground (n = 127)		*p*-Value
		n ^1^	% ^2^	n ^1^	% ^2^	n ^1^	% ^2^	
Age group (yrs)	18–35	78	32.6	42	37.5	36	28.3	0.041
	36–45	92	38.5	39	34.8	53	41.7	
	≥46	69	28.9	31	27.7	38	30.0	
Sex	Male	198	82.8	88	78.6	110	86.6	0.098
	Female	41	17.2	24	21.4	17	13.4	
Education	≤High school	143	59.8	72	64.3	71	55.9	0.182
	Tertiary	96	40.2	40	35.7	56	44.1	
Smoking status	Smoker	101	42.3	39	34.8	62	48.8	0.027
	Non-smoker	138	57.7	73	65.2	65	51.2	
Work experience	<5 years	84	35.1	48	42.9	36	28.3	0.015
	5–10 years	79	33.1	34	30.4	45	35.4	
	>10 years	76	31,8	30	26.8	46	36.2	

^1^ n: Frequency, ^2^ %: Percentage.

**Table 2:** Bivariate analysis of factors associated with chronic cough among mine workers’ chronic cough.

Variable	Category	OR ^1^	95% CI ^2^	*p*-Value
Mining type	Surface	1.00	–	–
	Underground	2.38	1.39–4.07	**0.002**
Smoking	Non-smoker	1.00	–	–
	Smoker	2.11	1.25–3.56	**0.005**
Dust exposure	Low	1.00	–	–
	Moderate	1.67	0.89–3.12	0.108
	High	3.45	1.83–6.49	**<0.001**
PPE ^3^ use	Consistent	1.00	–	–
	Inconsistent	1.94	1.15–3.27	**0.013**

^1^ OR: odds ratio, ^2^ CI: confidence interval, ^3^ PPE: personal protective equipment. Reference category indicated as “1”. Statistically significant associations (*p* < 0.05) are indicated in bold where applicable.

**Table 3:** Multivariable logistic regression analysis of chronic cough among mine workers.

Variable	Category	AOR ^1^	95% CI ^2^	*p*-Value
Mining type	Surface	1.00	–	–
	Underground	1.92	1.08–3.41	0.026
Smoking status	Non-smoker	1.00	–	–
	Smoker	1.78	1.02–3.11	0.041
Dust exposure	Low	1.00	–	–
	Moderate	1.41	0.73–2.72	0.305
	High	2.89	1.45–5.76	0.002
PPE ^3^ use	Consistent	1.00	–	–
	Inconsistent	1.63	0.94–2.83	0.081
Work experience	<5 years	1.00	–	–
	≥5 years	1.56	0.89–2.73	0.118

^1^ AOR: adjusted odds ratio, ^2^ CI: confidence interval, ^3^ PPE = personal protective equipment. The model was adjusted for age group, sex, education level, smoking status, work experience, dust exposure level, PPE use, ventilation quality, and exposure duration. Reference category indicated as “1”. Statistical significance was set at *p* < 0.05.

**Table 4:** Interaction between smoking and mining type on respiratory outcomes.

Variable	Category	AOR ^1^	95% CI ^2^	*p*-Value
Smoking × Underground	Yes	2.74	1.32–5.68	0.007
Smoking × Surface	Yes	1.45	0.72–2.91	0.298

^1^ AOR: adjusted odds ratio, ^2^ CI: confidence interval. Interaction terms represent the combined effect of smoking status and mining type on chronic cough. The reference group is non-smokers working in surface mining. Statistical significance was set at *p* < 0.05.

## Data Availability

The data underlying the findings of this study are available from the corresponding author upon reasonable request. Access to the dataset is subject to applicable ethical requirements and institutional data sharing policies.
